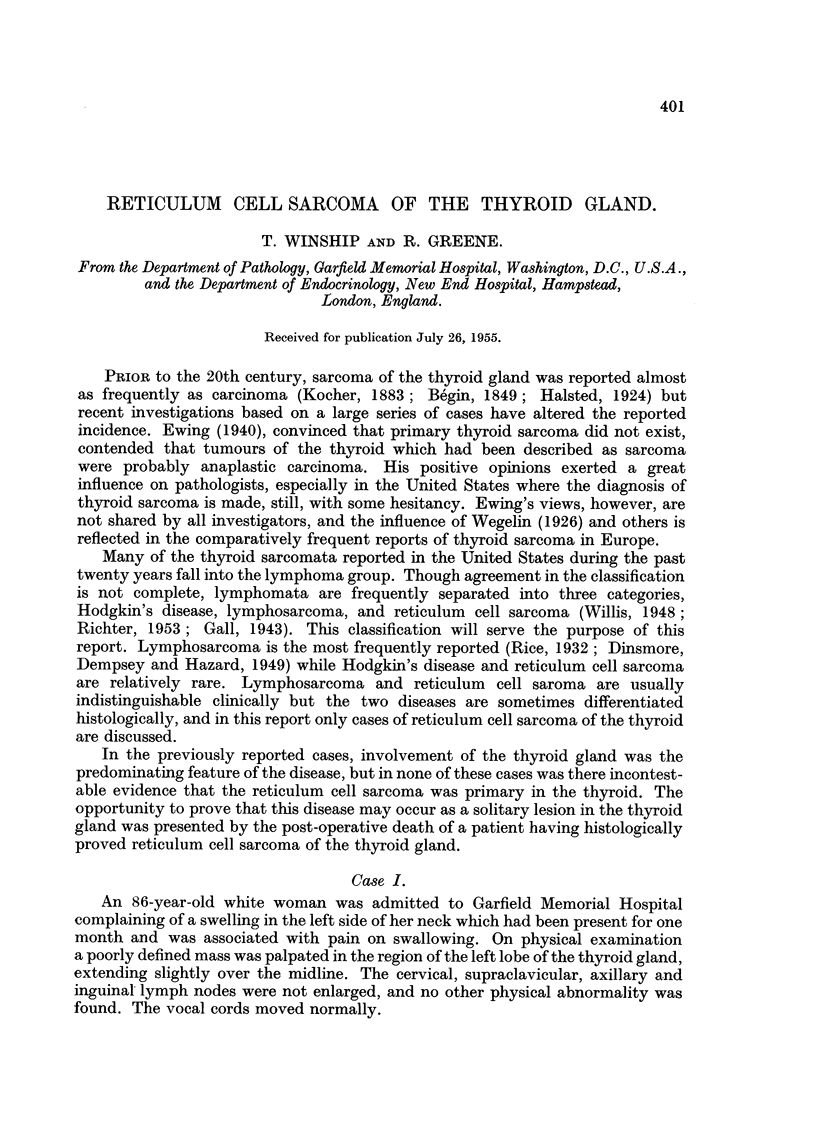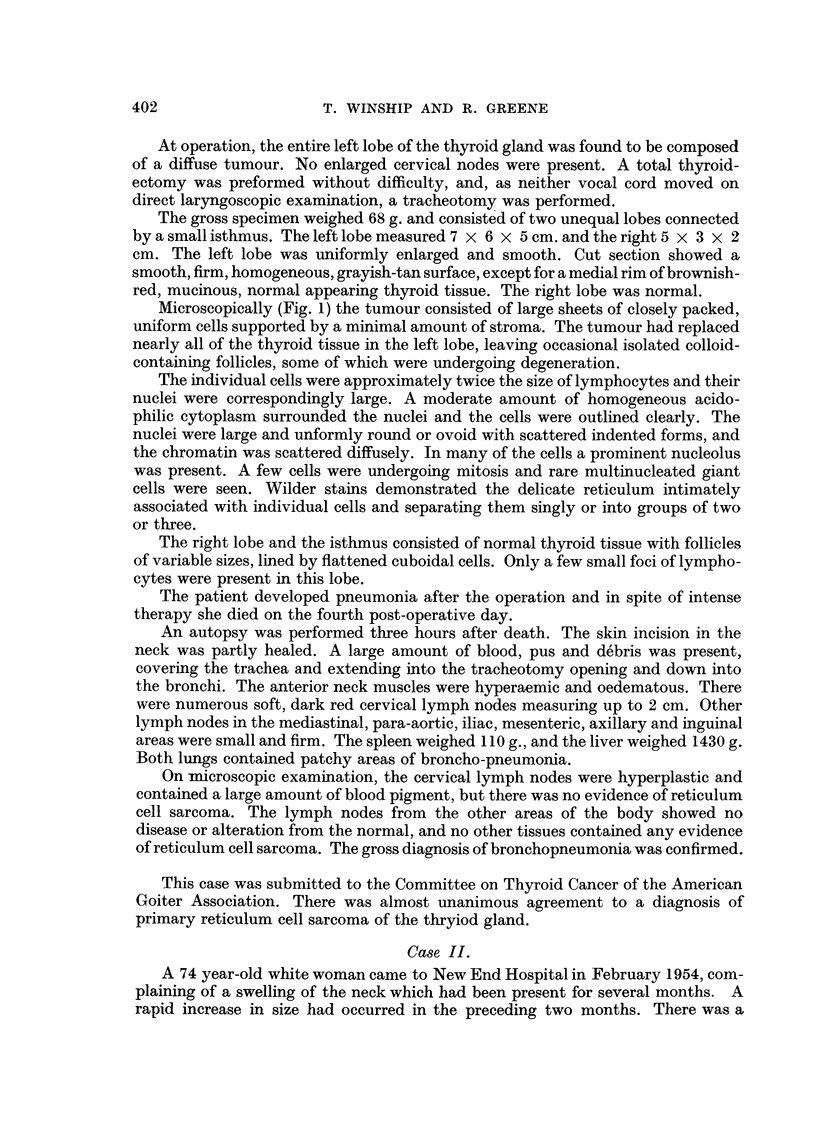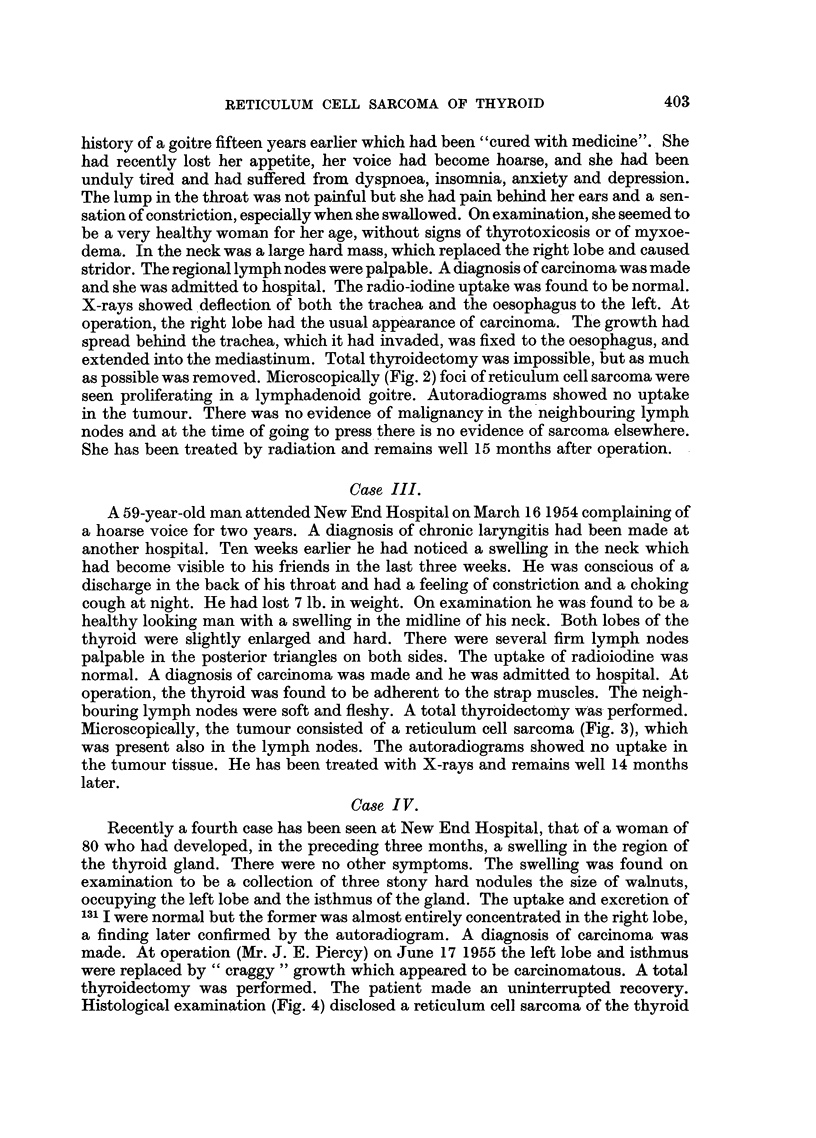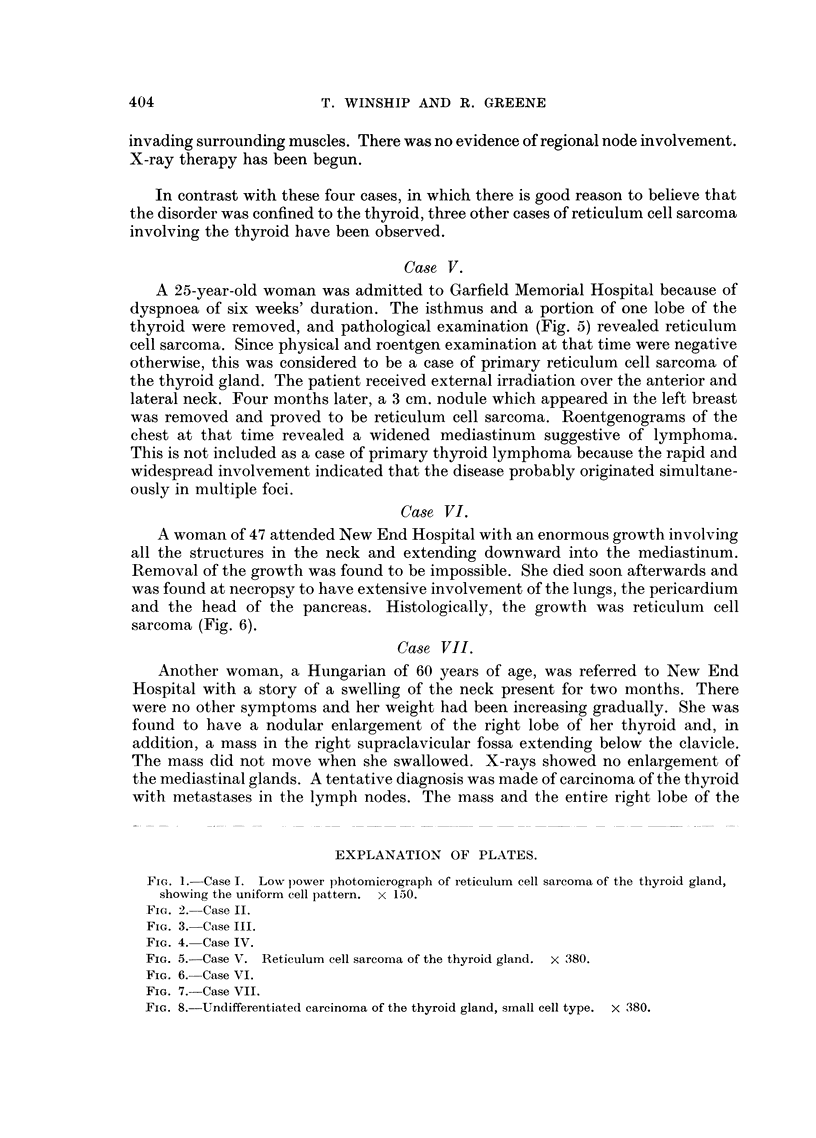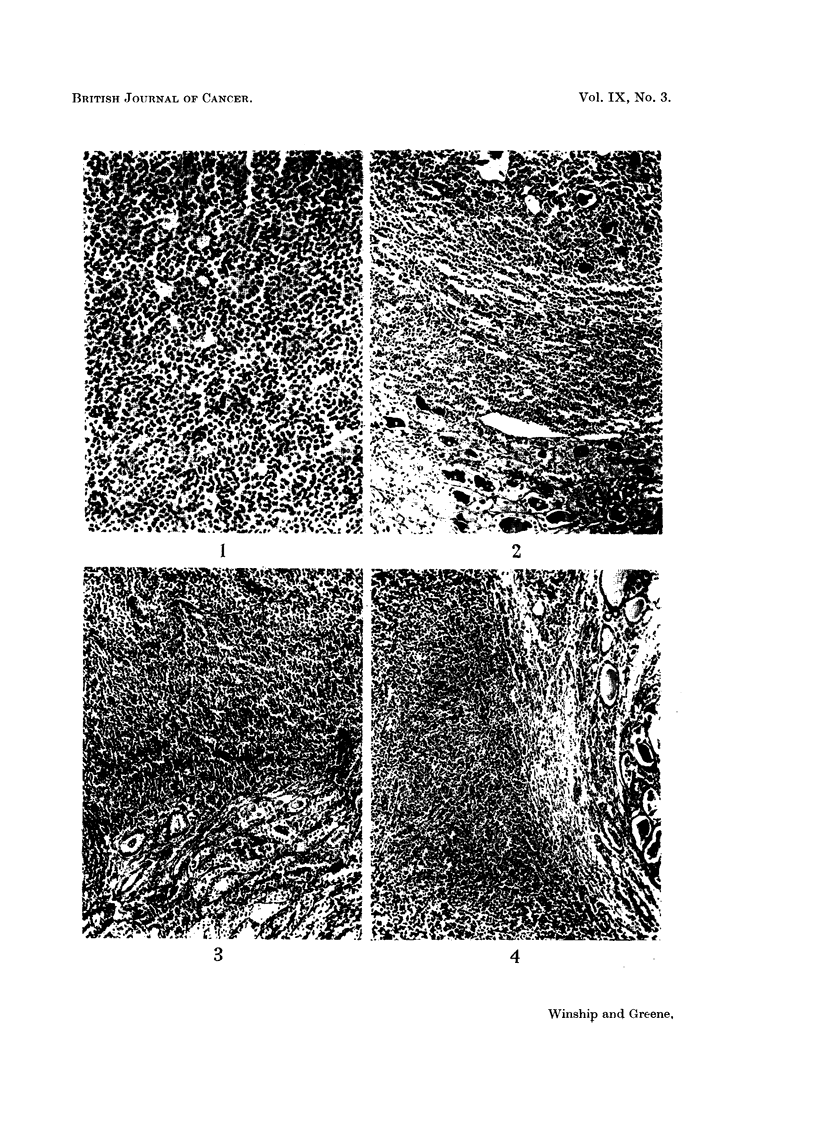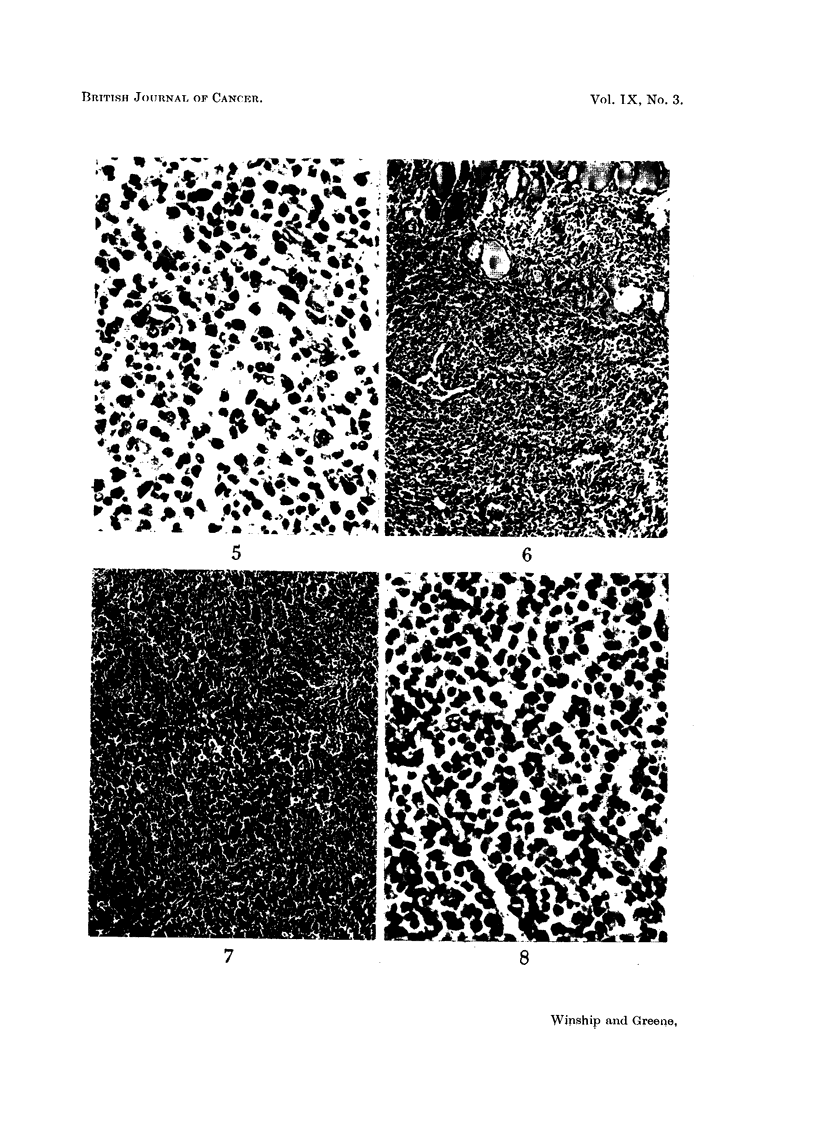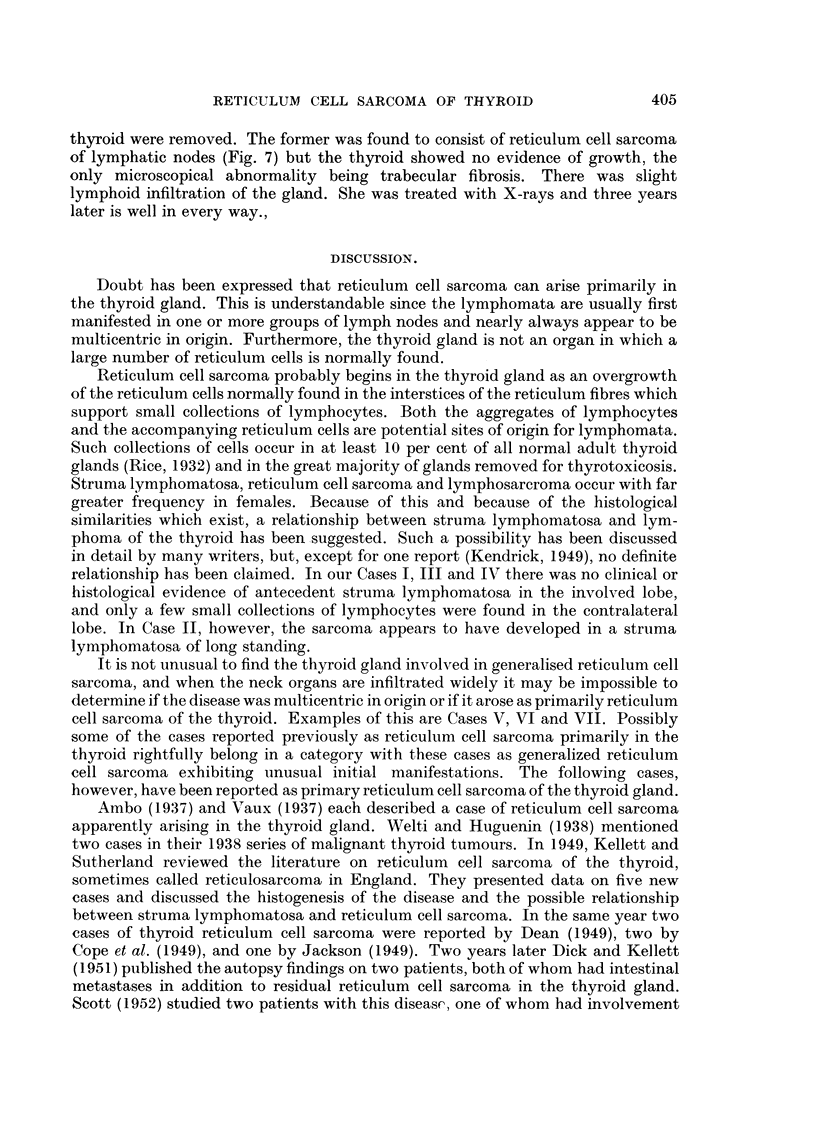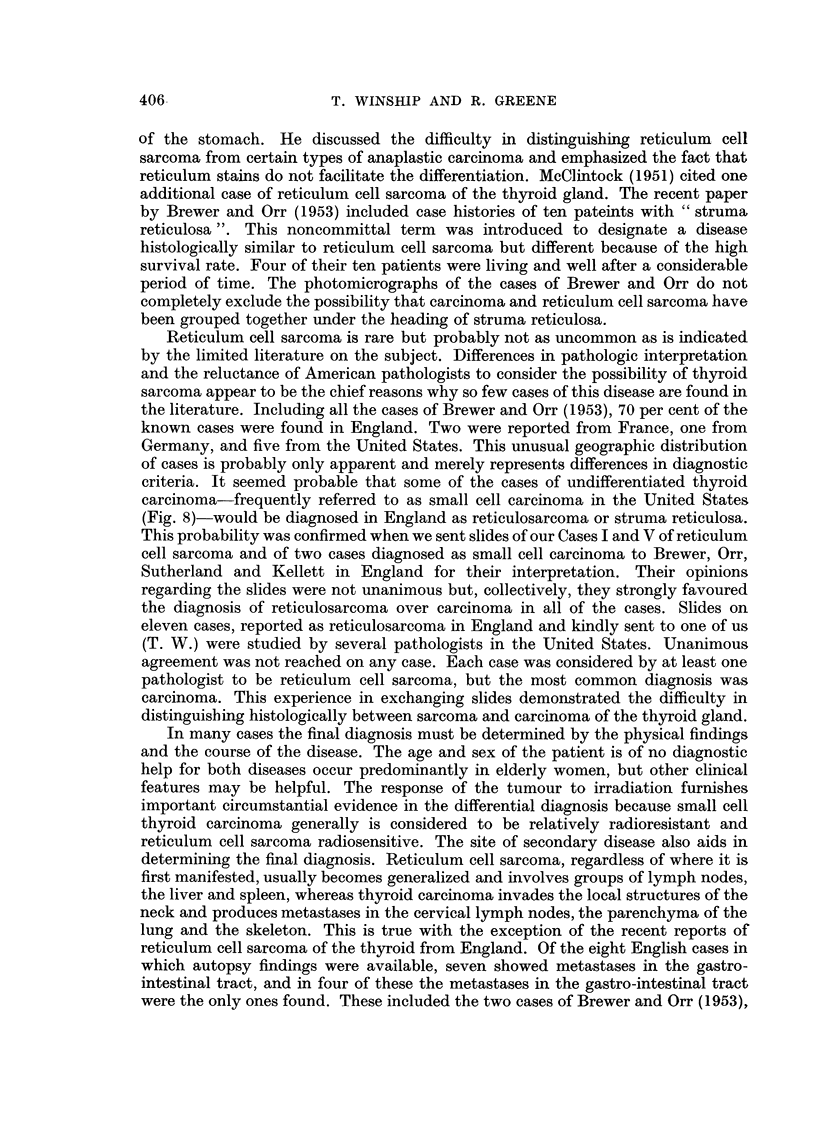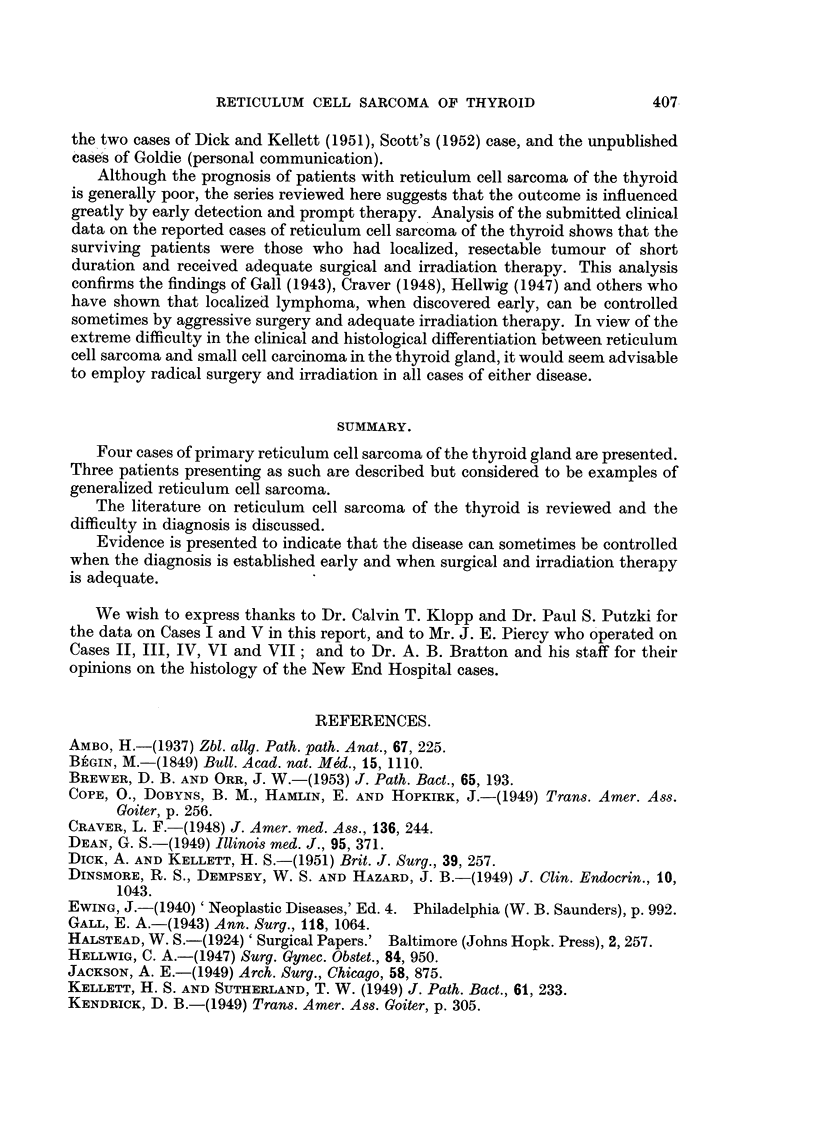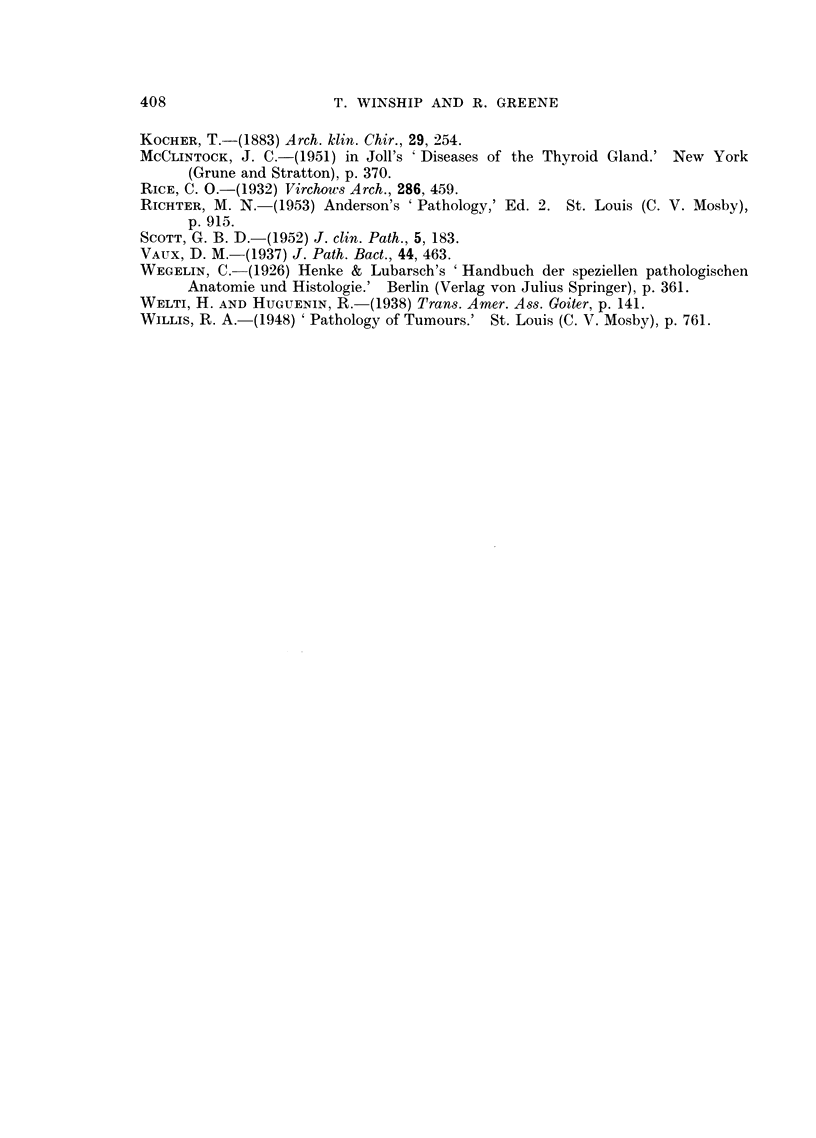# Reticulum Cell Sarcoma of the Thyroid Gland

**DOI:** 10.1038/bjc.1955.38

**Published:** 1955-09

**Authors:** T. Winship, R. Greene

## Abstract

**Images:**


### RETICULUM CELL SARCOMA OF THE THYROID GLAND.

T. WINSHIP AND R. GREENE.

*From the Department of Pathology, Garfield Memorial Hospital, Washington, D.C., U.S.A., and the Department of Endocrinology, New End Hospital, Hampstead, London, England.*

Received for publication July 26, 1955.

PRIOR to the 20th century, sarcoma of the thyroid gland was reported almost as frequently as carcinoma (Kocher, 1883; Bégin, 1849; Halsted, 1924) but recent investigations based on a large series of cases have altered the reported incidence. Ewing (1940), convinced that primary thyroid sarcoma did not exist, contended that tumours of the thyroid which had been described as sarcoma were probably anaplastic carcinoma. His positive opinions exerted a great influence on pathologists, especially in the United States where the diagnosis of thyroid sarcoma is made, still, with some hesitancy. Ewing's views, however, are not shared by all investigators, and the influence of Wegelin (1926) and others is reflected in the comparatively frequent reports of thyroid sarcoma in Europe.

Many of the thyroid sarcomata reported in the United States during the past twenty years fall into the lymphoma group. Though agreement in the classification is not complete, lymphomata are frequently separated into three categories, Hodgkin's disease, lymphosarcoma, and reticulum cell sarcoma (Willis, 1948; Richter, 1953; Gall, 1943). This classification will serve the purpose of this report. Lymphosarcoma is the most frequently reported (Rice, 1932; Dinsmore, Dempsey and Hazard, 1949) while Hodgkin's disease and reticulum cell sarcoma are relatively rare. Lymphosarcoma and reticulum cell saroma are usually indistinguishable clinically but the two diseases are sometimes differentiated histologically, and in this report only cases of reticulum cell sarcoma of the thyroid are discussed.

In the previously reported cases, involvement of the thyroid gland was the predominating feature of the disease, but in none of these cases was there incontestable evidence that the reticulum cell sarcoma was primary in the thyroid. The opportunity to prove that this disease may occur as a solitary lesion in the thyroid gland was presented by the post-operative death of a patient having histologically proved reticulum cell sarcoma of the thyroid gland.

### *Case I.*

An 86-year-old white woman was admitted to Garfield Memorial Hospital complaining of a swelling in the left side of her neck which had been present for one month and was associated with pain on swallowing. On physical examination a poorly defined mass was palpated in the region of the left lobe of the thyroid gland, extending slightly over the midline. The cervical, supraclavicular, axillary and inguinal lymph nodes were not enlarged, and no other physical abnormality was found. The vocal cords moved normally.

At operation, the entire left lobe of the thyroid gland was found to be composed of a diffuse tumour. No enlarged cervical nodes were present. A total thyroidectomy was preformed without difficulty, and, as neither vocal cord moved on direct laryngoscopic examination, a tracheotomy was performed.

The gross specimen weighed 68 g. and consisted of two unequal lobes connected by a small isthmus. The left lobe measured 7 × 6 × 5 cm. and the right 5 × 3 × 2 cm. The left lobe was uniformly enlarged and smooth. Cut section showed a smooth, firm, homogeneous, grayish-tan surface, except for a medial rim of brownish-red, mucinous, normal appearing thyroid tissue. The right lobe was normal.

Microscopically (Fig. 1) the tumour consisted of large sheets of closely packed, uniform cells supported by a minimal amount of stroma. The tumour had replaced nearly all of the thyroid tissue in the left lobe, leaving occasional isolated colloid-containing follicles, some of which were undergoing degeneration.

The individual cells were approximately twice the size of lymphocytes and their nuclei were correspondingly large. A moderate amount of homogeneous acidophilic cytoplasm surrounded the nuclei and the cells were outlined clearly. The nuclei were large and unformly round or ovoid with scattered indented forms, and the chromatin was scattered diffusely. In many of the cells a prominent nucleolus was present. A few cells were undergoing mitosis and rare multinucleated giant cells were seen. Wilder stains demonstrated the delicate reticulum intimately associated with individual cells and separating them singly or into groups of two or three.

The right lobe and the isthmus consisted of normal thyroid tissue with follicles of variable sizes, lined by flattened cuboidal cells. Only a few small foci of lymphocytes were present in this lobe.

The patient developed pneumonia after the operation and in spite of intense therapy she died on the fourth post-operative day.

An autopsy was performed three hours after death. The skin incision in the neck was partly healed. A large amount of blood, pus and débris was present, covering the trachea and extending into the tracheotomy opening and down into the bronchi. The anterior neck muscles were hyperaemic and oedematous. There were numerous soft, dark red cervical lymph nodes measuring up to 2 cm. Other lymph nodes in the mediastinal, para-aortic, iliac, mesenteric, axillary and inguinal areas were small and firm. The spleen weighed 110 g., and the liver weighed 1430 g. Both lungs contained patchy areas of broncho-pneumonia.

On microscopic examination, the cervical lymph nodes were hyperplastic and contained a large amount of blood pigment, but there was no evidence of reticulum cell sarcoma. The lymph nodes from the other areas of the body showed no disease or alteration from the normal, and no other tissues contained any evidence of reticulum cell sarcoma. The gross diagnosis of bronchopneumonia was confirmed.

This case was submitted to the Committee on Thyroid Cancer of the American Goiter Association. There was almost unanimous agreement to a diagnosis of primary reticulum cell sarcoma of the thryiod gland.

### *Case II.*

A 74 year-old white woman came to New End Hospital in February 1954, complaining of a swelling of the neck which had been present for several months. A rapid increase in size had occurred in the preceding two months. There was a history of a goitre fifteen years earlier which had been "cured with medicine". She had recently lost her appetite, her voice had become hoarse, and she had been unduly tired and had suffered from dyspnoea, insomnia, anxiety and depression. The lump in the throat was not painful but she had pain behind her ears and a sensation of constriction, especially when she swallowed. On examination, she seemed to be a very healthy woman for her age, without signs of thyrotoxicosis or of myxoedema. In the neck was a large hard mass, which replaced the right lobe and caused stridor. The regional lymph nodes were palpable. A diagnosis of carcinoma was made and she was admitted to hospital. The radio-iodine uptake was found to be normal. X-rays showed deflection of both the trachea and the oesophagus to the left. At operation, the right lobe had the usual appearance of carcinoma. The growth had spread behind the trachea, which it had invaded, was fixed to the oesophagus, and extended into the mediastinum. Total thyroidectomy was impossible, but as much as possible was removed. Microscopically (Fig. 2) foci of reticulum cell sarcoma were seen proliferating in a lymphadenoid goitre. Autoradiograms showed no uptake in the tumour. There was no evidence of malignancy in the neighbouring lymph nodes and at the time of going to press there is no evidence of sarcoma elsewhere. She has been treated by radiation and remains well 15 months after operation.

*Case III.*

A 59-year-old man attended New End Hospital on March 16 1954 complaining of a hoarse voice for two years. A diagnosis of chronic laryngitis had been made at another hospital. Ten weeks earlier he had noticed a swelling in the neck which had become visible to his friends in the last three weeks. He was conscious of a discharge in the back of his throat and had a feeling of constriction and a choking cough at night. He had lost 7 lb. in weight. On examination he was found to be a healthy looking man with a swelling in the midline of his neck. Both lobes of the thyroid were slightly enlarged and hard. There were several firm lymph nodes palpable in the posterior triangles on both sides. The uptake of radioiodine was normal. A diagnosis of carcinoma was made and he was admitted to hospital. At operation, the thyroid was found to be adherent to the strap muscles. The neighbouring lymph nodes were soft and fleshy. A total thyroidectomy was performed. Microscopically, the tumour consisted of a reticulum cell sarcoma (Fig. 3), which was present also in the lymph nodes. The autoradiograms showed no uptake in the tumour tissue. He has been treated with X-rays and remains well 14 months later.

*Case IV.*

Recently a fourth case has been seen at New End Hospital, that of a woman of 80 who had developed, in the preceding three months, a swelling in the region of the thyroid gland. There were no other symptoms. The swelling was found on examination to be a collection of three stony hard nodules the size of walnuts, occupying the left lobe and the isthmus of the gland. The uptake and excretion of $^{131}$I were normal but the former was almost entirely concentrated in the right lobe, a finding later confirmed by the autoradiogram. A diagnosis of carcinoma was made. At operation (Mr. J. E. Piercy) on June 17 1955 the left lobe and isthmus were replaced by " craggy " growth which appeared to be carcinomatous. A total thyroidectomy was performed. The patient made an uninterrupted recovery. Histological examination (Fig. 4) disclosed a reticulum cell sarcoma of the thyroid invading surrounding muscles. There was no evidence of regional node involvement. X-ray therapy has been begun.

In contrast with these four cases, in which there is good reason to believe that the disorder was confined to the thyroid, three other cases of reticulum cell sarcoma involving the thyroid have been observed.

*Case V.*

A 25-year-old woman was admitted to Garfield Memorial Hospital because of dyspnoea of six weeks' duration. The isthmus and a portion of one lobe of the thyroid were removed, and pathological examination (Fig. 5) revealed reticulum cell sarcoma. Since physical and roentgen examination at that time were negative otherwise, this was considered to be a case of primary reticulum cell sarcoma of the thyroid gland. The patient received external irradiation over the anterior and lateral neck. Four months later, a 3 cm. nodule which appeared in the left breast was removed and proved to be reticulum cell sarcoma. Roentgenograms of the chest at that time revealed a widened mediastinum suggestive of lymphoma. This is not included as a case of primary thyroid lymphoma because the rapid and widespread involvement indicated that the disease probably originated simultaneously in multiple foci.

*Case VI.*

A woman of 47 attended New End Hospital with an enormous growth involving all the structures in the neck and extending downward into the mediastinum. Removal of the growth was found to be impossible. She died soon afterwards and was found at necropsy to have extensive involvement of the lungs, the pericardium and the head of the pancreas. Histologically, the growth was reticulum cell sarcoma (Fig. 6).

*Case VII.*

Another woman, a Hungarian of 60 years of age, was referred to New End Hospital with a story of a swelling of the neck present for two months. There were no other symptoms and her weight had been increasing gradually. She was found to have a nodular enlargement of the right lobe of her thyroid and, in addition, a mass in the right supraclavicular fossa extending below the clavicle. The mass did not move when she swallowed. X-rays showed no enlargement of the mediastinal glands. A tentative diagnosis was made of carcinoma of the thyroid with metastases in the lymph nodes. The mass and the entire right lobe of the

EXPLANATION OF PLATES.

FIG. 1.—Case I. Low power photomicrograph of reticulum cell sarcoma of the thyroid gland, showing the uniform cell pattern. × 150.

FIG. 2.—Case II.

FIG. 3.—Case III.

FIG. 4.—Case IV.

FIG. 5.—Case V. Reticulum cell sarcoma of the thyroid gland. × 380.

FIG. 6.—Case VI.

FIG. 7.—Case VII.

FIG. 8.—Undifferentiated carcinoma of the thyroid gland, small cell type. × 380.

1

2

3

4

5 6

7 8

thyroid were removed. The former was found to consist of reticulum cell sarcoma of lymphatic nodes (Fig. 7) but the thyroid showed no evidence of growth, the only microscopical abnormality being trabecular fibrosis. There was slight lymphoid infiltration of the gland. She was treated with X-rays and three years later is well in every way.,

### DISCUSSION.

Doubt has been expressed that reticulum cell sarcoma can arise primarily in the thyroid gland. This is understandable since the lymphomata are usually first manifested in one or more groups of lymph nodes and nearly always appear to be multicentric in origin. Furthermore, the thyroid gland is not an organ in which a large number of reticulum cells is normally found.

Reticulum cell sarcoma probably begins in the thyroid gland as an overgrowth of the reticulum cells normally found in the interstices of the reticulum fibres which support small collections of lymphocytes. Both the aggregates of lymphocytes and the accompanying reticulum cells are potential sites of origin for lymphomata. Such collections of cells occur in at least 10 per cent of all normal adult thyroid glands (Rice, 1932) and in the great majority of glands removed for thyrotoxicosis. Struma lymphomatosa, reticulum cell sarcoma and lymphosarcroma occur with far greater frequency in females. Because of this and because of the histological similarities which exist, a relationship between struma lymphomatosa and lymphoma of the thyroid has been suggested. Such a possibility has been discussed in detail by many writers, but, except for one report (Kendrick, 1949), no definite relationship has been claimed. In our Cases I, III and IV there was no clinical or histological evidence of antecedent struma lymphomatosa in the involved lobe, and only a few small collections of lymphocytes were found in the contralateral lobe. In Case II, however, the sarcoma appears to have developed in a struma lymphomatosa of long standing.

It is not unusual to find the thyroid gland involved in generalised reticulum cell sarcoma, and when the neck organs are infiltrated widely it may be impossible to determine if the disease was multicentric in origin or if it arose as primarily reticulum cell sarcoma of the thyroid. Examples of this are Cases V, VI and VII. Possibly some of the cases reported previously as reticulum cell sarcoma primarily in the thyroid rightfully belong in a category with these cases as generalized reticulum cell sarcoma exhibiting unusual initial manifestations. The following cases, however, have been reported as primary reticulum cell sarcoma of the thyroid gland.

Ambo (1937) and Vaux (1937) each described a case of reticulum cell sarcoma apparently arising in the thyroid gland. Welti and Huguenin (1938) mentioned two cases in their 1938 series of malignant thyroid tumours. In 1949, Kellett and Sutherland reviewed the literature on reticulum cell sarcoma of the thyroid, sometimes called reticulosarcoma in England. They presented data on five new cases and discussed the histogenesis of the disease and the possible relationship between struma lymphomatosa and reticulum cell sarcoma. In the same year two cases of thyroid reticulum cell sarcoma were reported by Dean (1949), two by Cope *et al.* (1949), and one by Jackson (1949). Two years later Dick and Kellett (1951) published the autopsy findings on two patients, both of whom had intestinal metastases in addition to residual reticulum cell sarcoma in the thyroid gland. Scott (1952) studied two patients with this disease, one of whom had involvement of the stomach. He discussed the difficulty in distinguishing reticulum cell sarcoma from certain types of anaplastic carcinoma and emphasized the fact that reticulum stains do not facilitate the differentiation. McClintock (1951) cited one additional case of reticulum cell sarcoma of the thyroid gland. The recent paper by Brewer and Orr (1953) included case histories of ten pateints with " struma reticulosa ". This noncommittal term was introduced to designate a disease histologically similar to reticulum cell sarcoma but different because of the high survival rate. Four of their ten patients were living and well after a considerable period of time. The photomicrographs of the cases of Brewer and Orr do not completely exclude the possibility that carcinoma and reticulum cell sarcoma have been grouped together under the heading of struma reticulosa.

Reticulum cell sarcoma is rare but probably not as uncommon as is indicated by the limited literature on the subject. Differences in pathologic interpretation and the reluctance of American pathologists to consider the possibility of thyroid sarcoma appear to be the chief reasons why so few cases of this disease are found in the literature. Including all the cases of Brewer and Orr (1953), 70 per cent of the known cases were found in England. Two were reported from France, one from Germany, and five from the United States. This unusual geographic distribution of cases is probably only apparent and merely represents differences in diagnostic criteria. It seemed probable that some of the cases of undifferentiated thyroid carcinoma—frequently referred to as small cell carcinoma in the United States (Fig. 8)—would be diagnosed in England as reticulosarcoma or struma reticulosa. This probability was confirmed when we sent slides of our Cases I and V of reticulum cell sarcoma and of two cases diagnosed as small cell carcinoma to Brewer, Orr, Sutherland and Kellett in England for their interpretation. Their opinions regarding the slides were not unanimous but, collectively, they strongly favoured the diagnosis of reticulosarcoma over carcinoma in all of the cases. Slides on eleven cases, reported as reticulosarcoma in England and kindly sent to one of us (T. W.) were studied by several pathologists in the United States. Unanimous agreement was not reached on any case. Each case was considered by at least one pathologist to be reticulum cell sarcoma, but the most common diagnosis was carcinoma. This experience in exchanging slides demonstrated the difficulty in distinguishing histologically between sarcoma and carcinoma of the thyroid gland.

In many cases the final diagnosis must be determined by the physical findings and the course of the disease. The age and sex of the patient is of no diagnostic help for both diseases occur predominantly in elderly women, but other clinical features may be helpful. The response of the tumour to irradiation furnishes important circumstantial evidence in the differential diagnosis because small cell thyroid carcinoma generally is considered to be relatively radioresistant and reticulum cell sarcoma radiosensitive. The site of secondary disease also aids in determining the final diagnosis. Reticulum cell sarcoma, regardless of where it is first manifested, usually becomes generalized and involves groups of lymph nodes, the liver and spleen, whereas thyroid carcinoma invades the local structures of the neck and produces metastases in the cervical lymph nodes, the parenchyma of the lung and the skeleton. This is true with the exception of the recent reports of reticulum cell sarcoma of the thyroid from England. Of the eight English cases in which autopsy findings were available, seven showed metastases in the gastro-intestinal tract, and in four of these the metastases in the gastro-intestinal tract were the only ones found. These included the two cases of Brewer and Orr (1953), the two cases of Dick and Kellett (1951), Scott's (1952) case, and the unpublished cases of Goldie (personal communication).

Although the prognosis of patients with reticulum cell sarcoma of the thyroid is generally poor, the series reviewed here suggests that the outcome is influenced greatly by early detection and prompt therapy. Analysis of the submitted clinical data on the reported cases of reticulum cell sarcoma of the thyroid shows that the surviving patients were those who had localized, resectable tumour of short duration and received adequate surgical and irradiation therapy. This analysis confirms the findings of Gall (1943), Craver (1948), Hellwig (1947) and others who have shown that localized lymphoma, when discovered early, can be controlled sometimes by aggressive surgery and adequate irradiation therapy. In view of the extreme difficulty in the clinical and histological differentiation between reticulum cell sarcoma and small cell carcinoma in the thyroid gland, it would seem advisable to employ radical surgery and irradiation in all cases of either disease.

### SUMMARY.

Four cases of primary reticulum cell sarcoma of the thyroid gland are presented. Three patients presenting as such are described but considered to be examples of generalized reticulum cell sarcoma.

The literature on reticulum cell sarcoma of the thyroid is reviewed and the difficulty in diagnosis is discussed.

Evidence is presented to indicate that the disease can sometimes be controlled when the diagnosis is established early and when surgical and irradiation therapy is adequate.

We wish to express thanks to Dr. Calvin T. Klopp and Dr. Paul S. Putzki for the data on Cases I and V in this report, and to Mr. J. E. Piercy who operated on Cases II, III, IV, VI and VII ; and to Dr. A. B. Bratton and his staff for their opinions on the histology of the New End Hospital cases.

### REFERENCES.

Ambo, H.—(1937) *Zbl. allg. Path. path. Anat.*, **67**, 225.
Bégin, M.—(1849) *Bull. Acad. nat. Méd.*, **15**, 1110.
Brewer, D. B. and Orr, J. W.—(1953) *J. Path. Bact.*, **65**, 193.
Cope, O., Dobyns, B. M., Hamlin, E. and Hopkirk, J.—(1949) *Trans. Amer. Ass. Goiter*, p. 256.
Craver, L. F.—(1948) *J. Amer. med. Ass.*, **136**, 244.
Dean, G. S.—(1949) *Illinois med. J.*, **95**, 371.
Dick, A. and Kellett, H. S.—(1951) *Brit. J. Surg.*, **39**, 257.
Dinsmore, R. S., Dempsey, W. S. and Hazard, J. B.—(1949) *J. Clin. Endocrin.*, **10**, 1043.
Ewing, J.—(1940) ' Neoplastic Diseases,' Ed. 4. Philadelphia (W. B. Saunders), p. 992.
Gall, E. A.—(1943) *Ann. Surg.*, **118**, 1064.
Halstead, W. S.—(1924) ' Surgical Papers.' Baltimore (Johns Hopk. Press), **2**, 257.
Hellwig, C. A.—(1947) *Surg. Gynec. Obstet.*, **84**, 950.
Jackson, A. E.—(1949) *Arch. Surg., Chicago*, **58**, 875.
Kellett, H. S. and Sutherland, T. W. (1949) *J. Path. Bact.*, **61**, 233.
Kendrick, D. B.—(1949) *Trans. Amer. Ass. Goiter*, p. 305.

KOCHER, T.—(1883) *Arch. klin. Chir.*, **29**, 254.
MCCLINTOCK, J. C.—(1951) in Joll's ' Diseases of the Thyroid Gland.' New York (Grune and Stratton), p. 370.
RICE, C. O.—(1932) *Virchows Arch.*, **286**, 459.
RICHTER, M. N.—(1953) Anderson's ' Pathology,' Ed. 2. St. Louis (C. V. Mosby), p. 915.
SCOTT, G. B. D.—(1952) *J. clin. Path.*, **5**, 183.
VAUX, D. M.—(1937) *J. Path. Bact.*, **44**, 463.
WEGELIN, C.—(1926) Henke & Lubarsch's ' Handbuch der speziellen pathologischen Anatomie und Histologie.' Berlin (Verlag von Julius Springer), p. 361.
WELTI, H. AND HUGUENIN, R.—(1938) *Trans. Amer. Ass. Goiter*, p. 141.
WILLIS, R. A.—(1948) ' Pathology of Tumours.' St. Louis (C. V. Mosby), p. 761.